# Morpho-physiological and biochemical characterization of African spider plant (*Gynandropsis gynandra* (L.) Briq.) genotypes under drought and non-drought conditions

**DOI:** 10.3389/fpls.2023.1197462

**Published:** 2023-08-17

**Authors:** Tinashe Chatara, Cousin Musvosvi, Aristide Carlos Houdegbe, Samson Zeray Tesfay, Julia Sibiya

**Affiliations:** ^1^ School of Agriculture, Earth and Environmental Sciences, University of KwaZulu-Natal, Pietermaritzburg, South Africa; ^2^ School of Agricultural Sciences and Technology, Chinhoyi University of Technology, Chinhoyi, Zimbabwe; ^3^ Genetics, Biotechnology and Seed Science Unit (GBioS), Laboratory of Crop Production, Physiology and Plant Breeding, Faculty of Agronomic Sciences, University of Abomey-Calavi, Abomey-Calavi, Benin

**Keywords:** drought tolerance, water regime, African spider plant, phenotyping, proline

## Abstract

The African spider plant (*Gynandropsis gynandra* (L.) Briq.) is a nutrient-dense, climate-resilient indigenous vegetable with a C4 carbon fixation pathway. Understanding African spider plant drought tolerance mechanisms is essential for improving its performance in water-stressed areas. The objective of this study was to evaluate the stress tolerance potential of African spider plant accessions based on thirteen morphological, physiological, and biochemical traits under three different water treatment regimes. Eighteen accessions were evaluated over two growing seasons in the greenhouse using a split-split plot design with four replications and three water treatment-regimes namely optimum (100% field capacity), intermediate drought (50% field capacity) and, severe drought (30% field capacity). The results revealed that water regime had a significant effect (*P<* 0.01) on the accessions for the traits studied. A significant reduction across most of the studied traits was observed under drought conditions. However, proline content in all the accessions significantly rose under drought conditions. The principal component analysis revealed a considerable difference in the performance of the 18 African spider plant accessions under optimum and drought stress conditions. Several morphological and physiological parameters, including days to 50% flowering (*r = 0.80*), leaf length (*r = 0.72*), net photosynthesis (*r = 0.76*) and number of leaves per plant (*r = 0.79*), were positively associated with leaf yield under drought conditions. Cluster analysis categorized the 18 accessions and 13 measured parameters into 4 clusters, with cluster-1 exhibiting greater drought tolerance for most of the studied traits, and cluster-4 having the most drought-sensitive accessions. Among the accessions tested, accessions L3 and L5 demonstrated excellent drought tolerance and yield performance under both conditions. As a result, these accessions were selected as candidates for African spider plant drought tolerance breeding programs. These findings will serve as the foundation for future studies and will aid in improving food and nutrition security in the face of drought.

## Introduction

African spider plant (*Gynandropsis gynandra* (L.) Briq.) is a member of the Cleomaceae family which is native to Sub-Saharan Africa (SSA). It is also widely referred to as spider flower, spider plant, cats’ whiskers, as well as African cabbage. The plant has multiple uses, which include human food and medicine, animal feed, and has crop protectant abilities. *G. gynandra* is an essential leafy vegetable for achieving food security for households in remote regions of several African countries including South Africa, Zimbabwe, Zambia, Kenya, Namibia and Botswana (Mbugua et al., 2011; Keatinge et al., 2015). African spider plant has been found naturally thriving in seven of South Africa’s nine provinces; KwaZulu-Natal, Free State, Gauteng, Mpumalanga and Northern Cape Limpopo, Northwest (Chataika et al., 2022; Moyo and Aremu, 2022).

Vitamins C, A, E, B1, B2, and B9 as well as minerals including iron, zinc, calcium, copper, potassium, magnesium, manganese, phosphorus, and sodium are abundant in the species’ leaves and surpass those in most exotic vegetables (Abukutsa-Onyango, 2005; Odhav et al., 2007; Uusiku et al., 2010; Singh et al., 2013; Houdegbe et al., 2022). The leaves of *G. gynandra* are also high in proteins and fatty acids (Mnzava, 1990; Van Der Walt et al., 2009), as well as essential amino acids (histidine, isoleucine, leucine, lysine, methionine, phenylalanine, threonine, valine). Furthermore, spider plant has a variety of health-promoting secondary metabolites, including flavonoids, terpenoids, tannins, glucosinolates, aldehydes, ketones, sesquiterpenes, and several other phenolic compounds (Neugart et al., 2017; Sogbohossou et al., 2020; Somers et al., 2020; Chataika et al., 2022) with various therapeutic applications (plant extracts, drugs, etc.). The species is a valuable resource for the pharmaceutical industry because its extracts have antimicrobial (fungi and bacteria), anthelmintic (Ajaiyeoba et al., 2001), antimalarial (Igoli et al., 2016), hepatoprotective (Lakshmi Narsimhulu et al., 2019), antiarthritic (Narendhirakannan et al., 2005), antioxidant, anti-inflammatory (Chandradevan et al., 2020), immunomodulatory (Kori et al., 2009), antinociceptive (Ghogare et al., 2009), anticancer (Bala et al., 2010), antidiabetic (Ravichandra et al., 2014), and vasodilatory (Runnie et al., 2004) properties. Improvement of this vegetable will thus help to combat malnutrition, promote health, and generate income for stakeholders such as pharmaceutical companies and local communities.

Drought stress has continuously posed a threat and has become a serious problem to agricultural production. Recent studies have revealed that the majority of rural families in sub-Saharan Africa (SSA) rely on agriculture for their livelihood and food, thus extended periods of drought in those areas can have a devastating effect on the families (Meze-Hausken, 2000; Mupangwa et al., 2011; Rusinamhodzi et al., 2012). Drought is an extreme climatic phenomenon that is a prevalent natural hazard that leads to water scarcity and eventually famines (Abaje., et al., 2014). In plants, drought stress has been found to have an adverse effect on the morphological, physiological, and biochemical characteristics which leads to low yields (Baroowa et al., 2016; Mohammadi Alagoz et al., 2022).

Due to its efficient drought avoidance, tolerance, and escape mechanisms (Cernansky, 2015; Mabhaudhi et al., 2019; Chatara et al., 2023), *G. gynandra* thrives under insufficient and untimely precipitation. However, the leafy vegetable has received insufficient research in the past, although it is fast gaining attention of researchers and policymakers (Gido et al., 2017). As an underutilized horticultural crop, there are no established varieties of the African spider plant, and the majority of accessions exist in the form of landraces, that is mixtures of genotypes with common local adaptation (Houdegbe et al., 2022). Genetic diversity of the African spider plant has been the focus of research as breeders aim to establish knowledge on the existing genetic diversity. Genetic diversity can be identified by morphological, physiological, and biochemical markers (Chakhchar et al., 2015; Madisa and Tshamekang, 1997).

Phenotyping continues to be a fundamental criterion for evaluating germplasm on the basis of drought adaptability and essential morpho-physiological and biochemical traits, together with yield and its components (Monneveux et al., 2012; Passioura, 2012). Conventional breeding methods have enhanced crop productivity considerably due to the use of such traits in both ideal and low rainfall conditions. Thus, studying drought-associated traits is extremely important in enhancing crops for drought tolerance. Earlier studies have been centered on identifying key morphological and physiological traits in *G. gynandra* under irrigated conditions (Masuka et al., 2012; Wasonga et al., 2015; Omondi et al., 2017; Kangai Munene et al., 2018; Adeka et al., 2019). However, studies on the effects of drought stress on *G. gynandra* genotypes for morphological, physiological, and biochemical traits under drought conditions are lacking. Such studies can be used to better understand the mechanisms involved in drought tolerance. Therefore, this study was conducted to investigate the effects of different drought stress levels on morphological, physiological, and biochemical traits in African spider plant genotypes. Two key aspects were addressed. Firstly, identifying and selecting genotypes that are tolerant and sensitive to drought based on morpho-physiological and biochemical characteristics. Secondly to identify traits that can be used as markers in identifying tolerant genotypes under drought stress conditions.

## Materials and methods

### Plant material

Eighteen African spider plant accessions originating from East Africa (5), West Africa (5), Southern Africa (4) and Asia (4) were evaluated in this study. The accessions were obtained from the gene bank of the University of Abomey-Calavi, Laboratory of Genetics, Biotechnology, and Seed Science (Republic of Benin); the World Vegetable Center; the Kenya Resource Center for Indigenous Knowledge (Kenya); and the University of Ouagadougou (Burkina Faso) (**Table 1**). The chosen accessions are grown mainly under rain-fed conditions by smallholder farmers and are frequently exposed to prolonged drought stress. These accessions were also selected based on germination percentage and ability to produce high leaf yield under optimum conditions.

**Table 1:** List of genotypes used for the study.

Genotype	Genebank of Origin*	Country of origin	Region
L1	KENRIK	Kenya	East Africa
L2	University of Ouagadougou	Burkina-Faso	West Africa
L3	GBioS/UAC	Benin	West Africa
L4	GBioS/UAC	Benin	West Africa
L5	GBioS/UAC	Togo	West Africa
L6	University of Ouagadougou	Burkina-Faso	West Africa
L7	World Vegetable Center	Thailand	Asia
L8	World Vegetable Center	Zambia	Southern Africa
L9	World Vegetable Center	South Africa	Southern Africa
L10	World Vegetable Center	Malaysia	Asia
L11	World Vegetable Center	Uganda	East Africa
L12	World Vegetable Center	Malaysia	Asia
L13	KENRIK	Kenya	East Africa
L14	World Vegetable Center	Uganda	East Africa
L15	LUANAR	Malawi	Southern Africa
L16	Otjiwarongo	Namibia	Southern Africa
L17	World Vegetable Center	Laos	Asia
L18	KENRIK	Kenya	East Africa

*KENRIK, Kenya Resource Centre for Indigenous Knowledge; LUANAR, Lilongwe University of Agriculture and Natural Resources; GBioS/UAC, Laboratory of Genetics, Biotechnology and Seed Science, University of Abomey-Calavi.

### Experimental design, growth conditions and agronomic practices

Two experiments were carried out in 2020 and 2021 seasons at the University of KwaZulu-Natal, School of Agricultural, Earth, and Environmental Sciences in Pietermaritzburg, South Africa (29°37’34.1”S and 30°24’14.3”E), in the Controlled Environment Research Unit (CERU). The 18 African spider plant accessions were evaluated for drought tolerance using morphological, physiological and biochemical traits under three moisture regimes i.e. severe stress (30% field capacity), moderate stress (50% field capacity), and well-watered (100% field capacity), defined based on the findings of Masinde et al. (2005).

The seeds were sown in October and February of 2020 and 2021 seasons, respectively. The experiment was performed in a split-split plot design with four replicates, with the two seasons comprising the main plot, water stress (optimum, mild-drought stress and severe-drought stress) making-up the sub-plots, and the 18 genotypes constituting the sub-sub plots. Under each water regime, the seeds of each accession were sown in dedicated seedling trays and nurtured into seedlings before transplanting into individual pots two weeks after sowing. During the first seven days after transplanting, the seedlings were irrigated to keep the soil moisture in the pots at 100% field capacity. All irrigations were done using an automated drip irrigation system. Drought stress was applied from the 8th day after transplanting by ceasing irrigation until the harvest maturity stage, which occurred 21 days after transplanting. Using the method outlined by Kesiime et al. (2016), the amount of water applied in the pots was calculated based on the field capacity (FC) of the potting mixture. Soil moisture was constantly checked using a HS2 and HS2P Hydro sense II soil moisture measurement system and a Campbell scientific CR1000 series data loggers (Campbell scientific, Logan, Utah, USA).

The pots used for the study were medium-sized and made of plastic, with a pot height of 28 cm, diameter of 30 cm, and capacity of 4.5 kg. Each plot consisted of three pots, with a single plant in each pot. The potting mix used was a weed free Gromor composted pine bark with a volume of 60 dm^3^. Fertilizer was applied in accordance with the properties of the Gromor Potting Mix. Fertilizer containing (N:P:K) (2:3:2) was applied to seedlings in the pots during the transplanting process through the basal application technique at 40 g pot^-1^. The average air temperature and relative humidity in the greenhouse were 24 ± 3 °C and 65.7 ± 2%, respectively.

### Data collection

Data for the following traits were collected from samples taken from 3 plants from each plot.

### Phenological characteristics

Days to 50% flowering (Fl) were recorded from planting date to the date when 50% of the plants initiate flowering.

### Physiological characteristics

The following physiological traits were assessed:

#### Relative water content

Fully expanded relatively young leaves from each treatment were collected to get a precise estimation of relative water content (Rwc). After meticulously drying the surface of the leaf with tissue paper, everything was covered in polythene bags and taken to the laboratory. The leaf samples were weighed to establish the fresh weight of the leaf (FW). Following that, the samples were placed in petri dishes containing distilled water and left in the dark for an entire night. The excess water from the leaves was wiped with blotting paper before measuring the turgid weight (TW). The leaves were then dried in an oven at 80°C for 24 h, and their dry weight (DW) was measured.

The following formula was used to calculate the relative water content (Rwc):


Equation (1) $$\left(\%\right)\;RWC=\frac{FW-DW\;}{TW-DW}\times \;\;100\;\;\;\;$$

Where FW = Sample of fresh leaf weight TW = Sample of turgid leaf weight and DW = Sample of dry leaf weight.

#### Leaf gas exchange parameters

During the growing season, the following parameters were taken three times: Net photosynthesis (Photo), transpiration rate (Trans, mmol m^–2^ s^–1^), and stomatal conductance (Cond, mol m^–2^ s^–1^) using a LI-6400XT Portable Photosynthesis System (Licor Bioscience, Inc. Lincoln, Nebraska, USA) equipped with an infrared gas analyzer (IRGA) interconnected to a leaf chamber fluorometer (LCF). The outward leaf $CO_{2}$ concentration ( $C_{a}$ ) and the constructed saturating photosynthetic active radiation (PAR) were set to 400 µmol ^-1^and $1000\;µmol{\;}^{-2}\;m^{-2}s{\;}^{-1}$ , respectively. The temperature of the leaves was kept constant at 25°C. The water flow rate and relative humidity were both held constant at  500 µmol and 43%, respectively. To avoid stomatal closure due to low air humidity, the cuvette’s leaf-to-air vapor pressure deficit was kept constant at at 1.7 *kPa*. Parameters were measured on the third half-formed leaf from the plant’s tip between 08.30 and 12.00 a.m. by attaching the leaf inside the sensor head. Measurements were taken from three plants in both non-stressed and drought-stressed conditions for each accession.

#### Chlorophyll content (Spad)

Chlorophyll content (Spad) was measured on flag leaves of all three plants in a plot using a Biobase portable chlorophyll meter (Biobase, Jinan, China).

#### Proline content

Proline content was determined in the Plant Physiology Laboratory at the University of KwaZulu-Natal, Pietermaritzburg, South Africa. During harvesting, ten fully expanded flag leaf samples were randomly selected from non-stressed and drought-stressed treatments for proline analysis. The samples were freeze-dried and kept at 74°C. To obtain 0.1 g, the dried leaf tissue was ground and weighed. Upon obtaining the 0.1 g, 10 ml of 3% aqueous sulfosalicylic acid were used to homogenize the 0.1 g of leaf tissue. Proline was extracted using the acid-ninhydrin method described by Bates et al. (1973). After heating the samples for 1 hour at 100°C, 5 ml of toluene was added. The absorbance of the proline extract in toluene at 520 nm was measured using a UV-1800 spectrophotometer (Shimadzu Corporation, Kyoto, Japan).

The concentration of proline was then calculated using the formula proposed by Bates et al. (1973).


Equation (2) $$\begin{array}{c} {}[(µ\text{g}\;\text{proline}/\text{ml}\;\times \;\text{toluene})/115.5µg/µ\text{mol}]/[\left(\text{g}\;\text{sample}\right)/5]=\\ µ\text{mol}\;\text{proline}/\text{g of fresh weight material}\; \end{array}$$

Where 115.5 is the molecular weight of proline.

#### Morphological characteristics

1. Plant height (Ph, cm) measured from the surface of the soil to the tip of the flower for the three plants in each plot.
2. Leaf length (Ll, cm) determined on fully expanded leaves by measuring the length per each leaf from the pointy part at one end to the point at which the leaf joins the stalk at the other end with a meter ruler. Four leaves per plant were used.
3. Leaf width (Lw, cm) measured on fully expanded leaves, was achieved by recording the longest extension of any two points on the blade edge perpendicular to the leaf length axis using a meter ruler.
4. Stem diameter (Sd, mm) was recorded on the thickest part of the stem using a digital Vernier caliper.

#### Yield and yield components

1. Leaf yield (Ly, g) was determined by weighing and summing-up all leaf harvests per plot. Harvesting of leaves was carried out from the 6^th^ – 9^th^ week after planting.
2. Number of leaves per plant (Nl) was determined by counting the total number of leaves per plant in each plot. All three plants were counted, and the mean was determined per plot.

### Data analysis

R-software version-4.1.1 was used to perform all statistical analysis for the study (R Core Team, 2021). The package *agricolae* was used to perform the analysis of variance (ANOVA). Fisher’s Least Significant Difference (LSD) was also used to compare the significance of the three water treatments at a probability of *P< 0.05*, and the results are presented in a boxplot created in R version 4.1.1 (R Core Team, 2021) using the *ggplot2* package. A hierarchical clustering heatmap showing the studied genotypes and traits were constructed using the R package *ComplexHeatmap.* To analyze the correlation matrix plot, the R package *corrplot* was used, whilst two R packages *FactoMineR* and *factoextra* were utilized to produce the principal component analysis (PCA) and the PCA biplot. Data for split-split plot design was analyzed using a linear mixed model based on the following statistical model:


Equation (3) $$\begin{array}{c} Y_{ijkl}=\;\mu +block_{i}+A_{j}+BlockA_{ij}+\overset{´}{B_{k}+}AB_{jk}\\ +BlockB_{ik}+c_{i}+AC_{jl}+BC_{kl\;}+\;ABC_{jkl}+\;e_{ijkl} \end{array}$$

Where $Y_{ijkl}=$ measurement of outcome variable, μ= overall mean, $block_{i}=\;$ random effect of block or replication, $\;A_{j=}\;$ fixed effect of factor A (main plot), $BlockA_{ij}$ = random interaction between the block or rep and factor A (main plot factor)- this is the error term for factor A - main plot error, $B_{k}$ = fixed effect of factor B - sub plot, $AB_{jk}$ = fixed interaction between factor A and factor B, $BlockB_{ik}$ = random interaction between the block or rep and factor B (sub plot factor) - this is the error term for factor B and the interaction between factor A and B -sub plot error, $c_{i}$ = fixed effect of your factor C-sub plot. $AC_{jl}$ = fixed interaction between factor A and factor C, $BC_{kl\;}$ = fixed interaction between factor B and factor C, $ABC_{jkl}$ = fixed interaction between factor A, factor B, and factor C, $e_{ijkl}$ = residual error — correct error term for Sub -Sub plot factor C, AC, BC, and ABC.

## Results

### Effect of accessions, environments, and water regimes on morpho-physiological and biochemical traits

**Figure 1:**
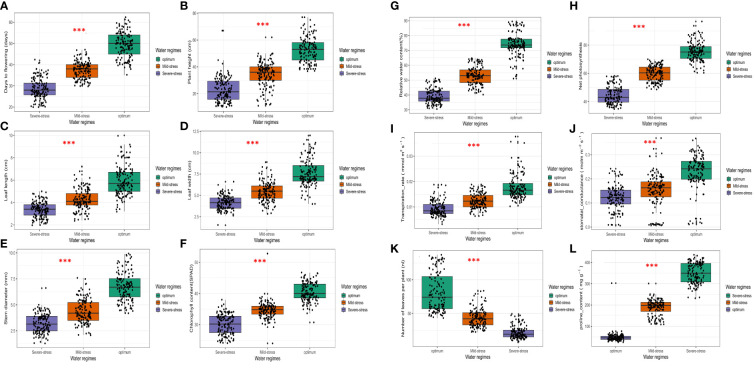
Boxplots showing distribution of morpho-physiological and biochemical traits recorded in 18 African spider plant accessions grown under three water regimes. ***P < 0.001, ns, non-significant. **(A)** Days to 50% flowering, **(B)** Plant height, **(C)** Leaf length, **(D)** Leaf width, **(E)** Stem diameter, **(F)** Chlorophyll content, **(G)** Relative water content, **(H)** Net photosynthesis rate, **(I)** Transpiration rate **(J)** Stomatal conductance, **(K)** Number of leaves per plant, **(L)** Proline content.

The ANOVA results provided in **Table 2** show the effects of growing season, water regime, and genotype factors, along with their interaction effects, and coefficients of variation (CVs) on the studied morpho-physiological and biochemical traits. The effect of planting season was not significant for most traits, except for Fl, Spad, and Cond (*P*< 0.05). Highly significant differences (*P*< 0.001) were recorded for the different water regimes for all traits (**Table 2**; **Figure 1**). The interaction between season and water regime was not significant for most traits except for Ph, Cond, Photo, Trans and Pro which were significantly affected (*P*< 0.001) (**Table 2**).

**Table 2:** *F*-values from a combined analysis of variance of the 13 traits of 18 African spider plant accessions under 3 water regimes and over two seasons.

Sources of variation	Df	Fl	Ph	Ll	Lw	Sd	Spad	Rwc	Cond	Photo	Trans	Nl	Ly	Pro
Season	1	9.01*	0.70ns	5.64ns	4.76ns	2.24ns	9.76*	0.31ns	6.17*	0.17ns	0.03ns	0.02ns	0.12ns	1.72ns
Rep (Season) = Error a	6	9.10***	8.29***	2.12ns	2.26*	5.25***	1.43ns	1.99ns	2.97**	16.75***	25.45***	3.56**	16.62***	7.68***
WR	2	198.71***	312.25***	166.28***	147.49***	232.06***	210.85***	12761.3***	61.06***	1170.40***	45.64***	2326.75***	1858.77***	611.23***
Season * WR	2	0.37ns	2.27ns	0.23ns	0.13ns	0.39ns	0.09ns	0.44ns	2.07ns	6.42*	2.35***	0.08ns	0.02ns	2.11ns
Rep(Season*WR) = Error b	12	5.91***	2.52**	3.01**	3.79***	2.70**	2.46**	1.17ns	2.85*	1.99*	7.31***	1.59ns	5.59***	4.67***
Gen	17	10.50***	14.56***	12.83***	9.21***	13.44***	2.31**	212.71***	4.02***	22.27***	3.49***	150.40***	467.16***	1.23ns
Season * Gen	17	0.84ns	2.63**	4.43***	2.37**	3.21***	0.75ns	0.44ns	2.32**	1.48ns	1.25ns	0.27ns	0.22ns	0.52ns
WR * Gen	34	2.11**	2.97***	3.64***	4.21***	2.07**	1.80**	45.73***	3.00***	4.48***	1.10ns	40.88***	164.33***	1.00ns
Season * WR * Gen	34	0.64ns	1.69*	2.99***	2.34***	1.37ns	1.17ns	0.36ns	1.96**	2.37***	1.10ns	0.49ns	0.24ns	0.74ns
CV		9.51	17.54	15.60	15.60	18.11	7.79	3.13	30.90	6.38	21.16	12.26	10.38	17.37

*P < 0.05, **P < 0.01, ***P < 0.001, ns, non-significant; Rep, Replication; WR, Water regime; Gen, Genotype; CV, Coefficient of variation; Df, Degree of freedom; Fl, Days to 50% flowering; Ph, Plant height; Ll, Leaf length; Lw, Leaf width; Sd, Stem diameter; Spad, Chlorophyll content; Rwc, Relative water content; Photo, Net photosynthesis rate; Cond, Stomatal conductance; Trans, Transpiration rate; Nl, Number of leaves per plant; Ly, Leaf yield; Pro, Proline content.

**Figure 2:**
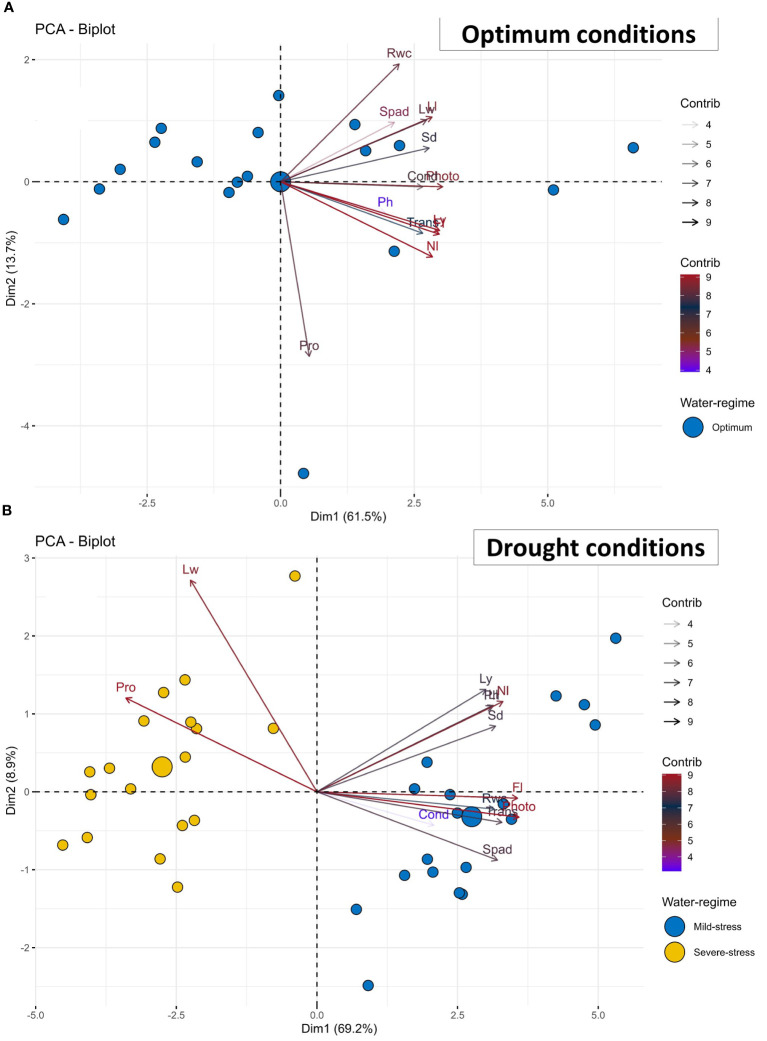
Principal component analysis (PCA)-biplot of 18 African Spider plant accessions based on the variance in 13 morpho-physiological and biochemical traits grown under **(A)** optimum and **(B)** drought conditions. Fl, Days to 50% flowering; Ph, Plant height; Ll, Leaf length; Lw, Leaf width; Sd, Stem diameter; Spad, Chlorophyll content; Rwc, Relative water content; Photo= Net photosynthesis rate; Cond, Stomatal conductance; Trans, Transpiration rate; Nl, Number of leaves per plant; Ly, Leaf yield; Pro, Proline content.

The accessions were significantly different for most studied traits (*P*< 0.001), except for Pro (**Table 2**). Season by genotype interaction was not significant for most traits, except for Ph, Ll, Lw and Sd (*P*< 0.001) (**Table 2**). Except for Trans and Pro where the interaction was not significant, the interaction between genotype and water regime was highly significant (*P*< 0.001) on all other traits studied (**Table 2**). Most of the traits had non-significant interaction effects of season, water regime and genotype, except for Ph, Ll, Lw, and Photo which were significant (**Table 2**).

### Performance of the accessions under different water regimes

**Supplementary Table 1** shows the mean values, and LSD values for comparing the accessions for morpho-physiological and biochemical traits under non-stressed and drought-stressed conditions. When compared to optimum conditions, drought stress reduced morpho-physiological traits (**Supplementary Table 1** and **Figure 1**). A phenological trait, Fl showed significant genotypic differences (*P*< 0.05) across the three conditions (**Table 2**), and was reduced as the stress intensified (**Figure 1A**). Drought stress considerably reduced Fl by 11 days under moderate stress and 20 days under severe stress conditions. Accessions L3 and L5 showed the most Fl under optimum and severe stress conditions. Additionally accessions L8 and L5 recorded the most Fl under mild stress conditions.

Across all three water regimes, Ph showed significant genotypic differences (*P*< 0.05) (**Table 2**), and ranged from 44.26 cm to 61.64 cm under optimum conditions, 18.41- 44.46 cm under mild stress conditions and 14.25-33.71 cm under severe stress (**Supplementary Table 1** and **Figure 1B**). Drought stress significantly reduced the Ph by 34.7% under mild stress and 56.63% under severe stressed conditions. Accessions L5 and L8 recorded the tallest plants whilst accessions L16 and L18 recorded the lowest Ph under optimum conditions. The highest Ph was observed in accessions L8 and L3, whereas accessions L12 and L18 recorded the lowest Ph under mild stress. Under severe stressed conditions, accessions L12, L16, and L13 recorded the lowest Ph. In parallel accessions L6 and L8 had the tallest plants.

Leaf length (Ll) significantly differed among the spider plant accessions (*P*< 0.05) (**Table 2**) across all three water regimes. Drought stress reduced Ll by 43.6% under severe stress and 28.3% under mild stress conditions (**Supplementary Table 1** and **Figure 1C**). Ll ranged from 4.74-8.11 cm under optimum conditions, 3.58-5.61 cm under mild stress and 2.64-4.01 cm under severe stressed conditions. Accessions L3 and L5 recorded the highest Ll under optimum whereas accessions L18 and L10 recorded the lowest leaf length. Under mild stress conditions, accessions L3 and L17 had the highest observed Ll measurements. Nonetheless, accessions L16 and L11 recorded the lowest Ll under mild stress. Contrastingly, accessions L3 and L14 had the highest leaf lengths under severe stress whereas accessions L2, L12 and L16 recorded the lowest leaf lengths under severe stress.

There were significant (*P*< 0.05) genotypic differences in Lw **Table 2**). Drought stress reduced Lw by 29.1% in mild stress conditions and 45.54% in severe stress conditions (**Supplementary Table 1** and **Figure 1D**). The range for Lw was 6.39-10.20 cm under optimum conditions, 3.96-7.16 cm under mild stress and 3.31-4.76 cm under severe stress. Under optimum conditions, accessions L3 and L5 recorded the highest Lw whereas accessions L1 and L18 recorded the lowest Lw. Accessions L3 and L8 had the highest leaf widths, whilst L11, L12 and L4 recorded the lowest Lw widths under mild stress. Accessions L15 and L9 recorded the highest Lw under severe stress but accessions L11 and L12 had the lowest Lw.

Genotypic differences were significant (*P*< 0.05) for stem diameter (**Table 2**). Water stress reduced Sd by 35% in mild stress conditions and by 52.6% in severe stress conditions (**Supplementary Table 1** and **Figure 1E**). Sd ranged from 4.86-9.00 mm under optimum conditions, 3.13- 6.24 mm under mild stress 2.24-4.05 mm under severe stress conditions. Accessions L3 and L5 recorded the highest Sd and the lowest Sd were observed for accessions L1 and L18 under optimum conditions. Under mild stress conditions, L18 and L1 were the lowest whilst accessions L3 and L8 recorded the highest Sd. The lowest recorded Sd under severe stress were for accessions L16 and L18 and the highest was observed for accessions L3 and L11.

Spad was significantly reduced by drought stress, with a reduction of 14.4% under mild stress and 15.5% under severe stress conditions (**Supplementary Table 1** and **Figure 1F**). The genotypic differences were significant across all three water regimes (*P*< 0.05) (**Table 2**). Spad ranged from 37.78- 43.33 under optimum condition, 32.59- 36.88 under mild stress and 27.78-32.51 under severe stress conditions. The highest Spad readings were observed in accessions L14 and L17 under optimum, L14 and L8 under mild stress and accessions L14 and L11 under severe stress conditions. The lowest readings for Spad were observed for accessions L10 and L8 under optimum conditions, L16 and L1 under mild stress and accessions L12 and L2 under severe stress conditions.

With regards to Rwc, significant genotypic differences were observed across all water regimes (*P*< 0.05) (**Table 2**). A decrease in available water caused a significant reduction in the Rwc of the leaves by 28.5% under mild stress and 47.3% under severe stress conditions (**Supplementary Table 1** and **Figure 1G**). Rwc ranged from 56.75- 88.00% under optimum, 42.31- 62.93% under mild stress and 31.84- 49.16% under severe stress conditions. Accessions L3 and L5 recorded the highest Rwc across all three water regimes. The lowest readings in Rwc were noted for accessions L7 and L18 under optimum, L2 and L13 under mild stress and L1 and L12 under severe stress conditions.

Photo varied significantly (*P*< 0.05) among the African spider plant accessions studied (**Table 2**), with L3, and L5 having significantly higher Photo across all three water regimes (**Supplementary Table 1**). Accessions L10 and L16, accessions L6 and L18 and accessions L18 and L13 recorded the lowest Photo under optimum, mild stress and severe stress respectively. Drought stress reduced Photo by 20.6% under mild stress and 47.3% under severe stress conditions (**Supplementary Table 1** and **Figure 1H**). The ranges for Photo varied from 69.74- 89.08% under optimum, 54.82-65.15% under mild stress and 37.77-56.74 under severe stress conditions.

Across all water treatments, significant (*P*< 0.05) genotypic transpiration rates were observed (**Table 2**). Drought stress reduced the Trans by 47.1% under severe stress conditions and 30.3% under mild stress conditions (**Supplementary Table 1** and **Figure 1I**). The Trans ranged from 0.0082-0.0114 mmol m^–2^ s^–1^ under severe stress, 0.0099-0.0138 mmol m^–2^ s^–1^ under mild stress and 0.0146-0.0211 under optimum conditions. Accessions L18 and L15 had the lowest Trans under severe stress whereas the highest rates were observed on L5 and L9. Under mild stress the lowest Trans were found in accessions L15 and L12 whilst the highest rates were found in accessions L1 and L10. Accessions L3 and L5 had the highest Trans whilst L15 and L18 had the lowest rates under optimum conditions (**Supplementary Table 1**).

For Cond, accessions L3 and L14, had significantly higher conductance under optimum conditions (**Supplementary Table 1**), L16 and L14 under mild stress and L3 and L17 under severe stress conditions. The genotypic differences were significant across all three water regimes (*P*< 0.05) (**Table 2**). Drought reduced Cond by 35% under mild stress and 47.5% under severe stress (**Supplementary Table 1** and **Figure 1J**). In terms of range, Cond ranged from 0.1625-0.2931 mol m^–2^ s^–1^ under optimum, 0.0866-0.2050 mol m^–2^ s^–1^ under mild stress and 0.1008-0.1703 mol m^–2^ s^–1^ under severe stress (**Supplementary Table 1**).

Water stress decreased the Nl by 53.1% and 72.8.4% under mild and severe stress, respectively (**Supplementary Table 1** and **Figure 1K**). Significant (*P*< 0.05) genotypic differences were observed across all three water regimes (**Figures 1K**, **Table 2**). Nl ranged from 11- 44 under severe stress, 26-76 under mild stress and 52-131 under optimum conditions. Accessions L3 and L5 had the highest Nl under optimum and severe stress conditions. Accessions L3 and L14 had the highest Nl under mild stress conditions. Accessions L2 and L12, L18 and L12 together with accessions L10, L12 and L13 had the least Nl under severe stress, mild stress, and optimum conditions respectively.

There were no significant genotypic differences among the genotypes for Pro (**Table 2**; **Supplementary Table 1**). However, Pro varied significantly across water regimes (**Table 2** and **Figure 1L**) and its concentration increased significantly as the stress intensified.

**Figure 3:**
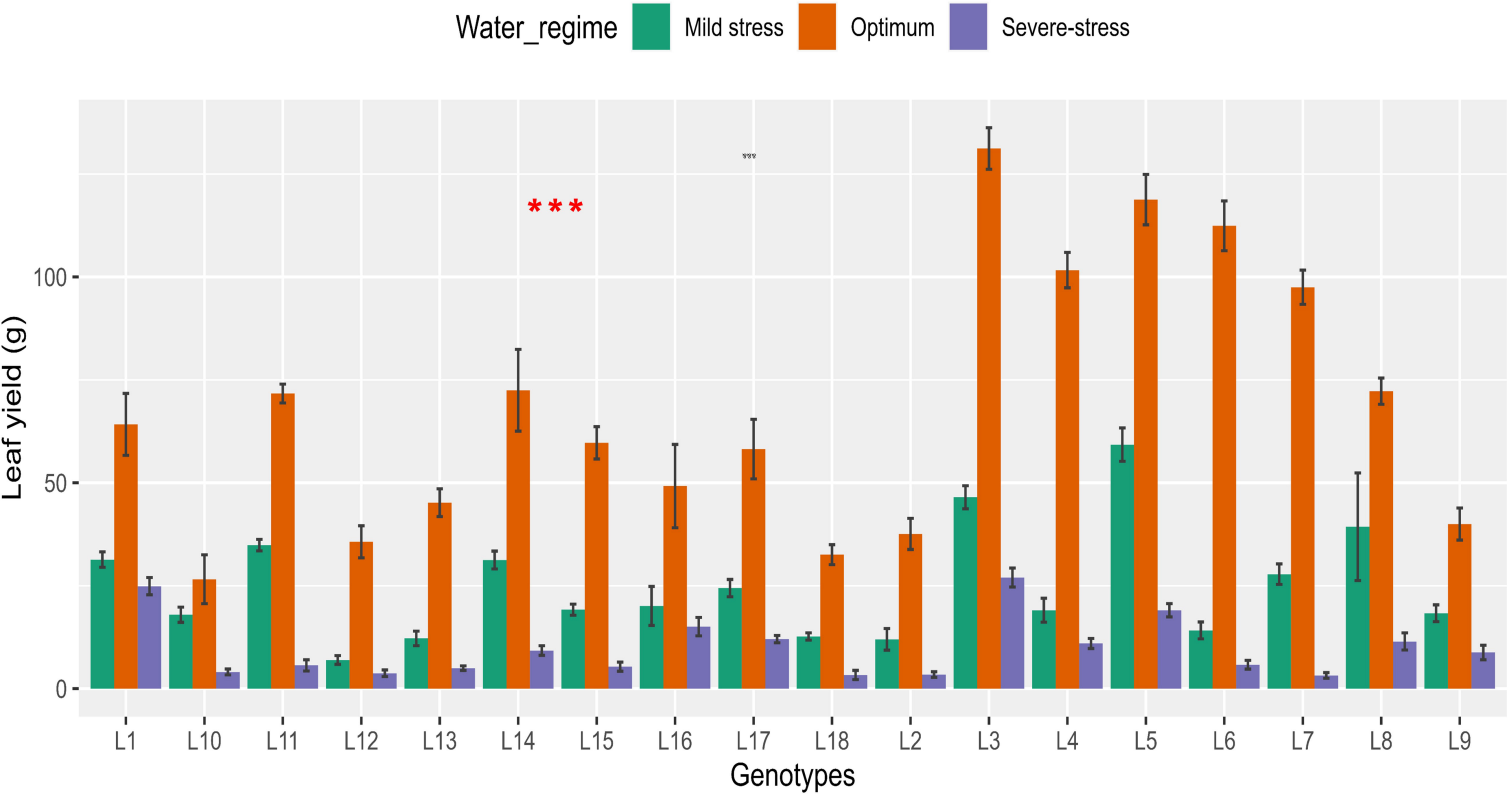
Barplots showing variation in leaf yield recorded in 18 African spider plant accessions grown under three water regimes. *** *P* < 0.001.

Water stress decreased Ly by 63.5% and 85.4% under mild and severe stress, respectively (**Supplementary Table 1**). There were significant (*P*< 0.05) genotypic differences observed across all water regimes (**Table 2**; **Figure 3**). Ly ranged from 3.33-26.99 g per plot under severe stress, 11.98-59.27 g per plot under mild stress and 26.57-131.19 g per plot under optimum conditions. Accessions L3 and L5 produced the highest Ly in all three water regimes. Accessions L18 and L2 had the lowest Ly under severe stress conditions. Accessions L2 and L13 under mild stress and accessions L10 and L18 under optimum produced the lowest Ly.

### Principal component analysis

**Table 3** presents the principal component analysis (PCA) results showing the proportion of total variance explained, and cumulative variance of studied morpho-physiological and biochemical traits among African spider plant accessions under non-stressed and drought-stressed conditions. Three principal components (PCs) with a cumulative variance of 82% were identified under optimum conditions. PC1 positively related to leaf yield, Fl, Photo, and Nl, accounting for 61% of total variation. Pro and Ph were negatively correlated with PC2. In contrast, Rwc was positively correlated with PC2, which was responsible for 14% of total variation. Ph and Spad positively correlated with PC3, which explained 7% of total variation.

**Table 3:** Rotated component matrix of 13 morpho-physiological and biochemical traits of 18 African spider plant accessions under drought-stressed and optimum conditions.

	Optimum			Drought-Stressed		
Traits	PC1	PC2	PC3	PC1	PC2	PC3
Fl	**0.32043**	-0.19588	-0.21092	-0.33774	-0.1781	0.232332
Ph	0.211481	-0.11642	**0.686428**	-0.31664	0.07903	-0.38559
Ll	**0.30578**	0.242105	-0.04807	-0.31816	**0.364343**	0.004674
Lw	0.294836	0.231222	-0.05752	-0.2421	**0.428673**	-0.12701
Sd	0.299911	0.126289	-0.06238	-0.33257	-0.04518	-0.23727
Spad	0.229639	0.222319	**0.499979**	-0.07015	**0.528818**	-0.09657
Rwc	0.239282	**0.440847**	0.120608	-0.23018	-0.03766	**0.321672**
Photo	**0.327278**	-0.01867	-0.2026	-0.32746	-0.31684	0.138791
Cond	0.286774	-0.01922	-0.25965	-0.13662	-0.2583	-0.3314
Trans	0.286964	-0.19249	-0.15048	-0.24614	-0.25001	0.113058
Nl	**0.306739**	-0.28105	0.167221	-0.0536	**0.319616**	**0.64286**
Pro	0.057781	**-0.653**	0.169809	-0.3664	0.102217	-0.13569
Ly	**0.321105**	-0.18197	-0.14963	-0.36568	-0.14215	0.196314
Explained variance (eigenvalue)	2.8278	1.3367	0.96179	8.990	1.154	0.812
Proportion of total variance (%)	61.512	13.744	7.11566	69.154	8.879	6.248
Cumulative variance (%)	61.512	75.25	82.37254	69.154	78.033	84.280

Fl, Days to 50% flowering; Ph, Plant height; Ll, Leaf length; Lw, Leaf width; Sd, Stem diameter; Spad, Chlorophyll content; Rwc, Relative water content; Photo= Net photosynthesis rate; Cond, Stomatal conductance; Trans, Transpiration rate; Nl, Number of leaves per plant; Ly, Leaf yield; Pro, Proline content. Bold values represent traits with the highest values that are positively related to that particular Principal component.

Similarly, three PCs with a cumulative variance of 84% were identified under drought-stress conditions. PC1 negatively correlated with all the thirteen studied and accounted for 69% of total variation. PC2 positively correlated with Lw, Spad, Ll, Nl and negatively correlated with net photosynthesis and stomatal conductance accounting for 9% of total variation. PC3 positively correlated with Nl, and Rwc accounting for 6% of total variation.

The association between African spider plant accessions and investigated traits is represented using principal component biplots under optimum and drought-stressed conditions (**Figure 2**). In terms of discriminating accessions, relatively small angles between dimension vectors indicated high trait correlation. Accessions that excelled at a specific trait were plotted closer and further away from the vector line. Under optimum conditions, the biplot was created using PC1 (61.5%) and PC2 (13.7%) (**Figure 2A**). The biplot results revealed that most traits clustered together in the biplot’s rightmost part region except for proline. However, most of the accessions were scattered at the leftmost part region of the biplot.

Under drought stressed conditions, the biplot was created using PC1 (69.2%) and PC2 (8.9%) (**Figure 2B**). The biplot findings confirmed that traits such as Lw and Pro were clustered together in the biplot’s leftmost region. The accessions under severe stress were clustered at the left most region whilst mild-stress accessions were clustered at the right. Traits such as Ly, Nl, Sd, Fl, Photo, Trans, Spad, Cond, Rwc, Ll and Ph were clustered to the right side.

### Pearson’s correlation coefficient analysis

**Figure 4:**
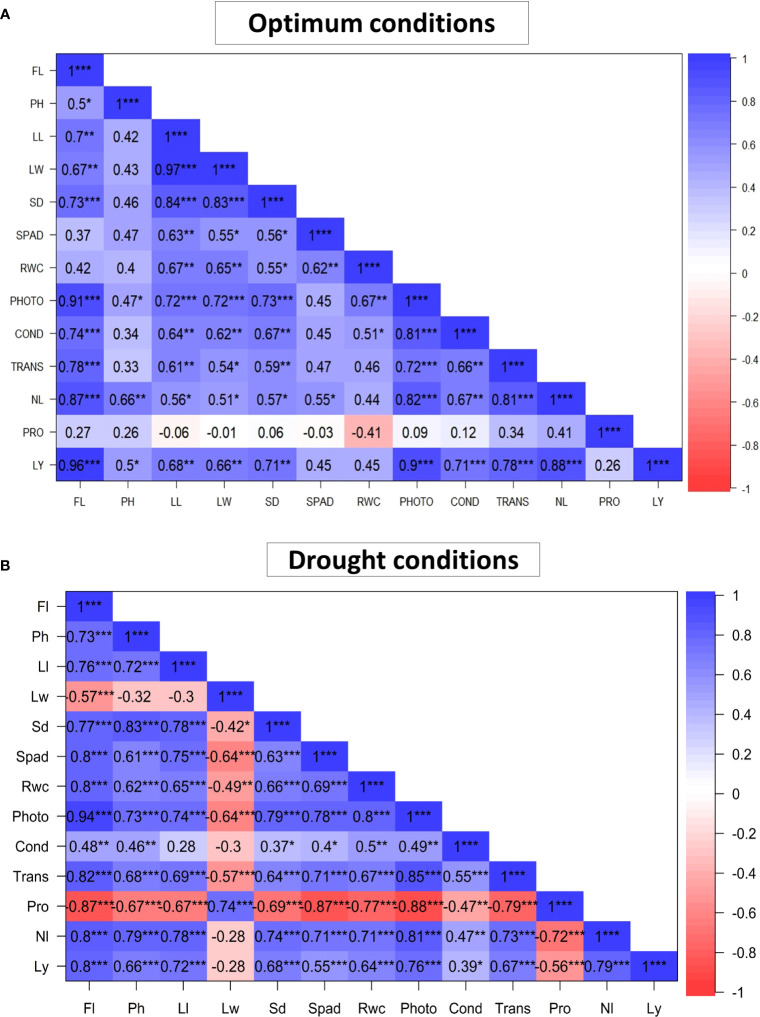
Pearson 's correlation coefficient of the 13 measured traits of 18 African Spider plant evaluated under optimum **(A)** and drought stress **(B)** conditions. * *P* < 0.05, ** *P* < 0.01, *** *P* < 0.001. Fl, Days to 50% flowering; Ph, Plant height; Ll, Leaf length; Lw, Leaf width; Sd, Stem diameter; Spad, Chlorophyll content; Rwc, Relative water content; Photo= Net photosynthesis rate; Cond, Stomatal conductance; Trans, Transpiration rate; Nl, Number of leaves per plant; Ly, Leaf yield; Pro, Proline content.

**Figure 4** illustrates Pearson correlation coefficients for the 13 characters studied. Under optimum conditions (**Figure 4A**), morphological traits such as Ll and Lw were positively and significantly correlated with each other (*r =* 0.97; *P< 0.001*). Ll was also positively and significantly correlated with Sd (*r =* 0.84; *P< 0.001*). Physiological traits were also found to be correlated with each other under optimum conditions. Photo was positively and significantly correlated with Trans (*r =* 0.72; *P< 0.001*) and Cond (*r =* 0.81*; P< 0.001*). Yield and yield components were found to be high and significantly correlated with each other. Ly was positively and significantly correlated with Nl (*r =* 0.88; *P< 0.001*). Ly was also positively and significantly correlated with Fl (*r =*0.96; *P< 0.001*), Ll (*r =*0.68; *P< 0.001*), Sd (*r =* 0.71; *P< 0.001*), Photo (*r =* 0.9; *P< 0.001*), Cond (*r =*0.71; *P< 0.01*), Trans (*r =* 0.78; *P< 0.001*) and Lw (*r =* 0.66; *P< 0.01*). Furthermore, there was a negative and non-significant correlation between Pro and Rwc (*r = -0.41; P = 0.09*).

In terms of correlation coefficients in drought conditions (**Figure 4B**), morphological traits such as Ll and Sd were positively and significantly correlated with each other (*r =* 0.78; *P< 0.001*). Physiological traits such as Spad were positively and significantly correlated with Trans (*r =* 0.71; *P< 0.001*), Photo (*r =* 0.78; *P< 0.001*) and Rwc (*r =* 0.69; *P< 0.001*). Ly was positively and significantly correlated with Fl (*r =* 0.80; *P< 0.001*), Ph (*r =* 0.66; *P< 0.001*), Ll (*r =* 0.72; *P< 0.001*), Sd (*r =* 0.68; *P< 0.001*), Rwc (*r =* 0.64; *P< 0.001*), Photo (*r =* 0.76; *P< 0.001*), Trans (*r =* 0.67; *P< 0.001*) and Nl (*r =* 0.79; *P< 0.001*). Pro was significantly and negatively correlated with all studied traits except for Lw (*r =* 0.74; P*< 0.001*), the only positively correlated trait with Pro.

### Cluster analysis

**Figure 5:**
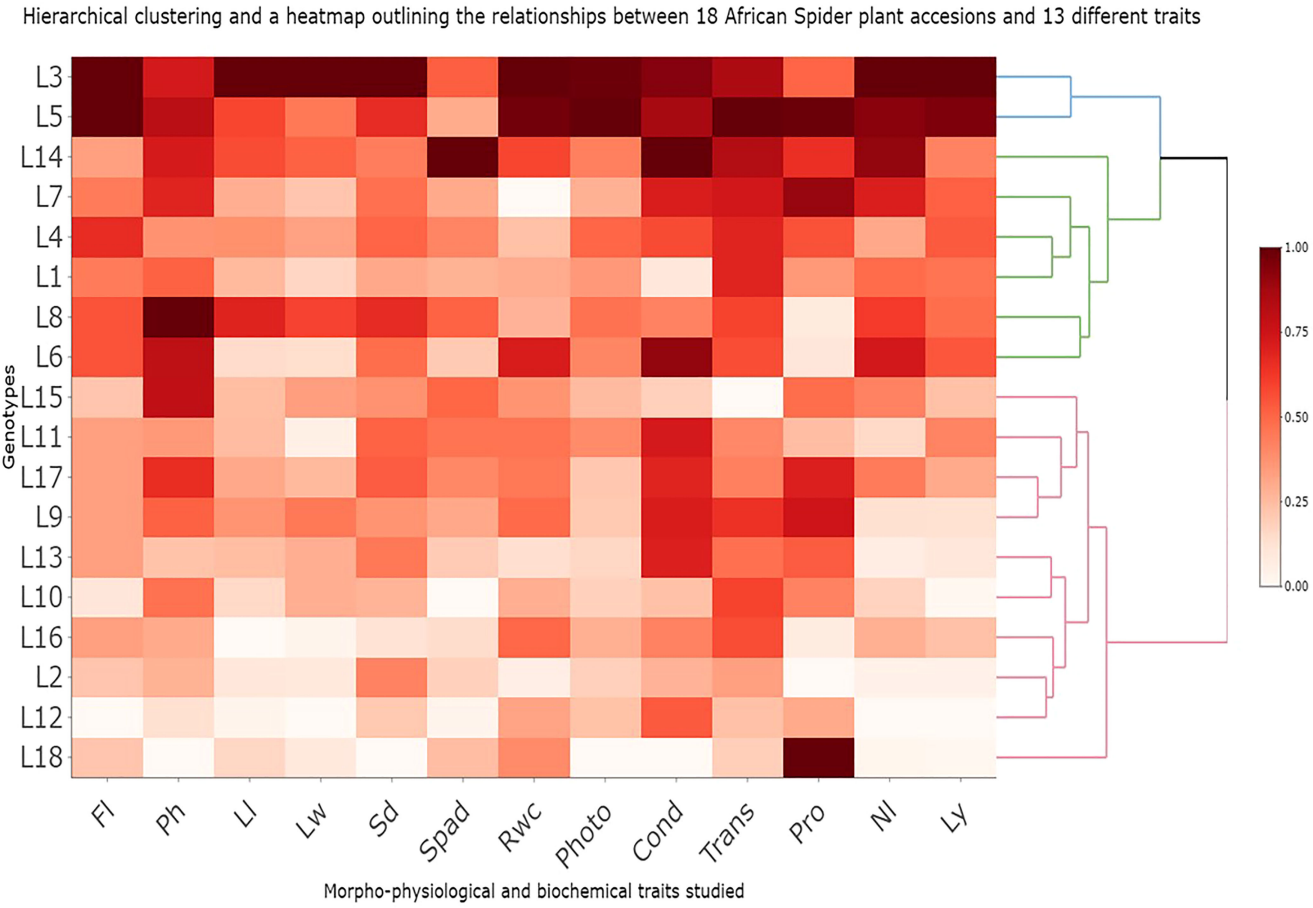
Hierarchical clustering and heatmap illustrating the associations among 18 African Spider plant and 13 different traits in respect to drought tolerance. FL, Days to 50% flowering; Ph, Plant height; Ll, Leaf length; Lw, Leaf width; Sd, Stem diameter; Spad, Chlorophyll content; Rwc, Relative water content; Photo= Net photosynthesis rate; Cond, Stomatal conductance; Trans, Transpiration rate; Nl, Number of leaves per plant; Ly, Leaf yield; Pro, Proline content.

A hierarchical clustering characterized by significant fold-change values through a complete method and Euclidean distance measurement was conducted to provide an overview of the morpho-physiological and biochemical traits and identify major clusters across 18 accessions under both control (optimum) and stress conditions (**Figure 5**). Based on cultivar-trait relationships, the various colors and intensities were adjusted. The lighter whitish-orange color represents lower values (drought-sensitive), whereas the darker red color represents higher values (drought-tolerant). Based on the cluster heatmap, four clusters were identified.

The 18 African Spider plant accessions were organized into four row-clusters, with cluster-1, cluster-2, cluster-3, and cluster-4 each consisting of 2, 1, 5 and 10 accessions, respectively, with the most closely related accessions within each cluster joining. Cluster 1 showed accessions with the highest drought tolerance based on leaf yield and other traits. Accessions L3 and L5 were found in cluster 1 thus representing genotypes with the highest drought tolerance. Cluster 2 showed a low level of tolerance with one accession L14 present in this cluster. Cluster 3 accessions showed a great degree of drought tolerance to drought stress with accessions L7, L4, L1, L8 and L6 representing this cluster. Cluster 4 had drought sensitive genotypes with 10 genotypes present in this cluster. Accessions L15, L11, L17, L9, L13, L10, L16, L2, L12, and L18 were all found in cluster 4.

## Discussion

The current study assessed the morpho-physiological and biochemical responses of African spider plant accessions to identify genotypes with a combination of traits that are adaptable to water-limited conditions. All the accessions used in the study were chosen from different pedigrees and most of the measured traits are quantitatively inherited and thus expected to be affected by the African spider plant genotypes, water regimes, and genotype by water regime interaction.

### Effect of accessions, environments, and water regimes on morpho-physiological and biochemical traits

The study observed variable responses amongst the accessions for the measured morpho-physiological and biochemical traits varied considerably across the African spider plant accessions. Water stress affected several phenological processes (**Supplementary Table 1** and **Figure 1**). For example, Fl a significant phenological trait linked to present photosynthesis and assimilate displacement from reserve pools in vegetative tissues, was reduced. Drought, in particular, reduces a plant’s life cycle and duration of flowering. The flowering period shortens under drought conditions due to increased leaf senescence, reduced photosynthetic activity, and sink limitation (Fang and Xiong, 2015; Shavrukov et al., 2017; Paudel et al., 2021). Moreover, the relatively short flowering period has a direct impact on leaf number and leaf size, which accounts for a large portion of the decrease in African spider plant yield. Early flowering is a drought escape mechanism that has been developed by plants to complete its life cycle under water deficit stress. This phenomenon explains why accessions L12, L2 and L18 recorded extremely low Ll, Lw, Nl and eventually very low Ly. Because the African spider plant is a facultative long-day plant, selecting for delayed bolting has possibility of increasing yield (Koevenig, 1973; Zorde et al., 2020). This phenomenon is backed by this study which showed that accessions L3 and L5 had more flowering days and because of that recorded the highest Nl and Ly.

Morphological traits such as Ph, Ll, Lw and Sd were significantly reduced by drought stress. Water stress has a significant impact on cell expansion and growth, which is linked with a loss of cell turgor, resulting in a reduction in Ph. Similar patterns have previously been reported in legumes (Baroowa and Gogoi, 2012). Reduced leaf water status impairs cell division in dehydrated plants, causing a decrease in leaf morphological traits (Dale, 1988; Tardieu, 2013).

Leaf gas exchange parameters were reduced under both severe and mild stress conditions. The decrease in photosynthetic rates was caused by both stomatal and non-stomatal factors. Drought stress has been shown to reduce photosynthesis in faba bean (Girma and Haile, 2014), grain legumes (Farooq et al., 2017), and dry bean (Lanna et al., 2017). Furthermore, under drought stress, stomata close, causing a decrease in stomatal conductance and, as a result, a lower photosynthetic rate (Reddy et al., 2004; Chaves et al., 2009). Drought stress causes a water shortage within the plant tissue, which significantly inhibits photosynthesis. It has been reported that stomatal closure reduces bean photosynthetic rates (Brestic et al., 1995). Berry and Bjorkman (1980) claim that an integration of stomatal and non-stomatal effects on photosynthesis occurs, based on the severity of drought stress. Tezara et al. (1999) concluded that water stress restricts photosynthesis by reducing the supply of ribulose-1,5-bisphosphate (RuBP) because of low ATP-synthesis.

Furthermore, under water deficit conditions, stomata begin to close, resulting in decreased stomatal conductance, which may lead to decreased photosynthetic rate (Jamshidi Zinab et al., 2022). Previous studies have shown that a decrease in stomatal conductance in drought-stressed plants reduces photosynthesis (Flexas et al., 2004; Kirschbaum, 2004; Reddy et al., 2004). Stomatal closure during water stress, as well as decreased CO_2_ availability in chloroplasts, are major causes of reduced photosynthetic activity (Franks et al., 2015). Stomatal closure restricts CO_2_ from entering the leaf and decreases photosynthetic carbon assimilation in favor of photorespiration. Drought stress has a similar effect on transpiration and photosynthetic rate (Li et al., 2017; Morales et al., 2020). When plants are stressed by drought, their stomata close, resulting in less transpiration and a restriction of gas exchange between the leaves and the environment.

Drought stress significantly reduced Spad, with a reduction of 14.4% under mild stress and 15.5% under severe stress conditions. The decrease in Spad could have stemmed from drought-damaged leaves turning yellowish. Drought stress has been shown to reduce chlorophyll content in wheat (Talebi, 2011), triticale (Mohammadi Alagoz et al., 2023), maize (Mohammadkhani and Heidari, 2007), chickpea (Mafakheri et al., 2010), soybean (Makbul et al., 2011), and rice (Jnandabhiram and Sailen Prasad, 2012). The reduction in Spad is due to chloroplast damage stemming from active oxygen species (Golldack et al., 2014). Drought stress causes the formation of reactive oxygen species (ROS) such as $O_{2}$ and H_2_O_2_, which destroy chlorophyll (Smironff, 1993; Foyer et al., 1994).

The Rwc estimates in the drought stressed and optimum treatments were consistent with the findings reported by Goodarzian Ghahfarokhi et al. (2015) and Soltys-Kalina et al. (2016). Drought stress reduced the Rwc of the leaves by 28.5% under mild stress and 47.3% under severe stress, but accessions differed in preserving their relative water content under both water stressed and optimum conditions. The water balance of a plant is interrupted during drought stress, resulting in a decrease in relative water content and water potential of leaves (Bogale et al., 2011). High relative water content values are widely regarded as an index of stress tolerance. Rwc is also thought to be a reliable predictor of the severity of water stress as evidenced by accessions L3 and L5 which showed a high Rwc value across all water treatment.

Osmotic adjustment at the cellular level is an important method for reducing the adverse effects of drought induced damage in crops (Kazemi Oskuei et al., 2023). As the stress increased, the proline content increased significantly. Plants can effectively protect cells from water stress by increasing proline accumulation and stabilizing osmotic potential with the external environment, as noted in wheat (Mwadzingeni et al., 2016). Elevated proline accumulation plays an adaptative role in conferring tolerance in plants (Bhaskara et al., 2015; Kazemi Oskuei et al., 2023). During drought stress, proline accumulation performs the function of a compatible solute, limiting water loss from plant cells (Li et al., 2019). It also aids in the supply of energy for plant survival and growth (Furlan et al., 2020). As a result, accumulation of proline can be employed as an effective selection criterion in germplasm screening studies for drought tolerance (Arteaga et al., 2020; Saddique et al., 2020; Belay et al., 2021).

Water stress causes defoliation and the cessation of new leaf production, resulting in fewer leaves. This explains why the Nl reduced significantly as the stress intensified from mild to severe stress. Selecting for higher yields under both stressed and non-stressed conditions allows accessions to retain high yield rankings because the same accessions will perform well in either situation.

The reported retention of high Ly under stressed and non-stressed conditions in some accessions, such as L3, and L5, supports Foulkes et al. (2007) findings that accessions doing well under non-stressed conditions retain high Ly under stress. However, the strong cross-over associations found in this study, on the other hand, were caused by severe stress imposed on the accessions, resulting in yield losses of approximately 63.5% and 85.4% under mild and severe stress respectively in comparison to stress imposed by Foulkes et al. (2007).

### Principal component analysis

In this study, a PCA was used to identify the most crucial morpho-physiological and biochemical traits for the distribution of a set of accessions in the three treatments (**Table 3**; **Figure 2**) and, as a result, the traits that may be most important for drought tolerance. PCA-biplot is a multivariate analytic technique that combines traits and variables in two or more dimensions while minimizing overlapping variations to make it simpler to discover key elements for selection (Kose et al., 2018; Huqe et al., 2021). Strong positive loading of Ly, Fl, Photo, and the Nl in the PC1 under optimum conditions indicates that they have a significant effect and can be selected for at the same time due to their direct influence on each other (**Table 3**). Under drought stressed conditions, PC2 had a favorable correlation with Nl and Ll, Spad and Lw. PCA results also showed that variables Lw and Pro clustered together in the PCA biplot, closely scattering around the accessions under severe stress conditions, indicating that their role in selecting best characters under severe stress conditions is critical.

### Pearson’s correlation coefficient and cluster analysis

Understanding the relationship between traits allows for effective and simultaneous selection. The moderate to high positive and significant correlations *(r >* 0.50) of Ly with Fl, Ph, Photo, Ll, Sd, Trans and Nl under both optimum and stressed conditions (**Figure 2**) suggest these characteristics make a direct contribution to yield and should be characterized as influential target traits during selection. Under optimum conditions, leaf yield was also positively and significantly correlated with Fl, Ll, Sd, Photo, Cond, Trans, Lw, and Nl which emphasizes the importance of those yield components in contributing to high leaf yield under optimum conditions. Previous research has found a positive and strong correlation between Ly and Ph, Sd, Ll and Lw (Zakaria et al., 2017). Similarly, Kangai Munene et al., 2018; Houdegbe et al., 2022 discovered a positive and strong relationship between Ph and Nl.

Under drought stressed conditions, Ly was positively and significantly correlated with Fl, Ph, Ll, Sd, Rwc, Photo, Trans and Nl. Such a positive correlation between these traits suggests that simultaneous and direct selection for these desired traits is feasible. Pro was significantly and negatively correlated with all studied traits except for Lw. Proline is an osmolyte that also functions as an osmotic regulator and has antioxidant activity by scavenging reactive oxygen species (ROS) and protecting plants from further oxidative damage and cell death (Upadhyay et al., 2020).Various studies have indicated that a higher unrestricted accumulation of proline is related with drought-resilience and lower content with drought sensitivity (Asao, 2012; Upadhyay et al., 2020).

Although Pro significantly increased in all African spider plant accessions studied when subjected to drought stress, the scale of accumulation differed, and the increase was observed on sensitive accessions such as L18. Corresponding research studies in wheat (Rampino et al., 2006) and maize (Ibarra-caballero et al., 1988) found that free proline levels multiplied as relative water content decreased in all sensitive wheat genotypes. Though proline levels rise during drought stress, the function of proline accumulation in conferring drought stress tolerance is debatable. As a result, more research is needed to avoid differences in opinion about whether an increase in proline levels can help plants cope with drought stress (Arteaga et al., 2020; Belay et al., 2021).

Ultimately, accessions in cluster 1 and, to a lesser extent, cluster 3 maybe valuable genetic stocks for breeding drought tolerance in African spider plant, considering the morpho-physiological traits. Accessions L3, L5 and L7, L4, L1, L8 and L6 from these clusters were superior for several traits. According to the current study’s findings, accessions such as L3, L5 and L7, L4, L1, L8 and L6, can maintain notable high leaf yields in both optimal and stressful conditions. The accessions’ tolerance response to drought stress is attributed primarily to their genetic constitution, which regulate the key traits in African Spider plant under drought stress which is consistent with previous research (Islam et al., 2007; Mohi-Ud-din et al., 2021; Chatara et al., 2023).

## Conclusion

In this study, we evaluated a panel of 18 African spider plant accessions based on 13 morpho-physiological, and biochemical character traits under three different water regimes. Overall, most traits studied were significantly affected by the season of study and accessions, whereas variability due to water regimes was significant for all traits studied. Significant changes in phenological, physiological, morphological, and yield traits were observed under the three different water regimes. Proline accumulates in response to stress, but when evaluated at a specific time point, proline may not be a reliable indicator or marker for indirect selection for water stress stressed yield. The present study also concluded that the investigated accessions contain valuable genetic diversity for drought tolerance. The drought-tolerant accessions reported in this study based on discriminative analyses, L3 and L5, could be highly suggested as promising parents for spider plant drought tolerance improvement breeding programs, along with developing stable and high-performing lines. Future research can also investigate the molecular facets of these promising inbred lines, such as the molecular mechanism and gene expression profile of candidate drought resistant genes.

## Data availability statement

The original contributions presented in the study are included in the article/**Supplementary Material**. Further inquiries can be directed to the corresponding authors.

## Author contributions

Conceptualization: TC, AH and JS. Data curation: TC. Formal analysis: TC, SZT and CM. Funding acquisition: JS. Investigation: TC. Methodology: TC and AH. Project administration: TC. Resources: JS. Supervision: JS. Validation: TC, CM, SZT and JS. Writing—original draft: TC and CM. Writing—review and editing: CM, AH and JS. All authors have read and agreed to the published version of the manuscript.
