# Supplementary material for: Morpho-physiological and biochemical characterization of African spider plant (Gynandropsis gynandra (L.) Briq.) genotypes under drought and non-drought conditions
Source: Front Plant Sci. 2023 Aug 17;14:1197462. doi: 10.3389/fpls.2023.1197462 (PMC10469808; doi:10.3389/fpls.2023.1197462)
Supplement: Supplementary file 1 [file DataSheet_1.docx]

Supplementary Material

Morpho-physiological and biochemical characterization of African spider plant (*Gynandropsis gynandra* (L.) Briq.) genotypes under drought and non-drought conditions

**Tinashe Chatara^1*^, Cousin Musvosvi^2^, Aristide Carlos Houdegbe^1,3^, Samson Tesfaye^1^ and Julia Sibiya^1*^**

*** Correspondence:** Tinashe Chatara: [chataratinashe@gmail.com](mailto:chataratinashe@gmail.com) and Julia Sibiya: [sibiyaj@ukzn.ac.za](mailto:sibiyaj@ukzn.ac.za)

.

Table 1a Means for days to 50% flowering, plant height, and leaf length of 18 African Spider plant accessions across three water regimes over two seasons.

|  | **Fl** | | | |  | **Ph** | | | |  | **Ll** | | | |
| --- | --- | --- | --- | --- | --- | --- | --- | --- | --- | --- | --- | --- | --- | --- |
| **Gen** | **SS** | **MS** | **WW** | **Mean** |  | **SS** | **MS** | **WW** | **Mean** |  | **SS** | **MS** | **WW** | **Mean** |
| L1 | 31 | 37 | 49 | **39** |  | 22.13 | 32.64 | 55.09 | **36.62** |  | 3.74 | 3.95 | 5.50 | **4.40** |
| L2 | 27 | 38 | 47 | **37** |  | 16.93 | 33.34 | 46.56 | **32.28** |  | 2.64 | 4.11 | 5.48 | **4.08** |
| L3 | 35 | 39 | 58 | **44** |  | 25.53 | 41.61 | 54.58 | **40.57** |  | 4.01 | 5.61 | 8.11 | **5.91** |
| L4 | 31 | 39 | 52 | **41** |  | 26.18 | 27.75 | 47.86 | **33.93** |  | 3.14 | 3.90 | 6.90 | **4.65** |
| L5 | 32 | 44 | 55 | **44** |  | 26.43 | 38.29 | 61.64 | **42.12** |  | 3.65 | 4.38 | 7.20 | **5.08** |
| L6 | 29 | 37 | 55 | **40** |  | 33.71 | 33.66 | 58.28 | **41.88** |  | 3.05 | 4.13 | 5.35 | **4.18** |
| L7 | 26 | 37 | 53 | **39** |  | 28.24 | 37.11 | 54.06 | **39.80** |  | 3.44 | 4.56 | 5.41 | **4.47** |
| L8 | 31 | 40 | 50 | **40** |  | 30.86 | 44.46 | 61.35 | **45.56** |  | 3.49 | 5.34 | 7.05 | **5.29** |
| L9 | 29 | 36 | 48 | **38** |  | 19.26 | 37.58 | 53.00 | **36.61** |  | 3.56 | 4.06 | 6.25 | **4.62** |
| L10 | 27 | 36 | 45 | **36** |  | 19.73 | 35.38 | 52.11 | **35.74** |  | 3.30 | 4.16 | 5.10 | **4.19** |
| L11 | 27 | 38 | 49 | **38** |  | 18.66 | 31.88 | 50.44 | **33.66** |  | 3.09 | 3.79 | 6.25 | **4.38** |
| L12 | 25 | 34 | 45 | **35** |  | 14.49 | 21.89 | 52.08 | **29.49** |  | 2.90 | 3.58 | 5.31 | **3.93** |
| L13 | 28 | 37 | 48 | **38** |  | 14.25 | 32.34 | 47.35 | **31.31** |  | 3.25 | 4.26 | 5.59 | **4.37** |
| L14 | 27 | 37 | 50 | **38** |  | 26.79 | 41.30 | 53.30 | **40.46** |  | 3.93 | 4.59 | 6.61 | **5.04** |
| L15 | 26 | 37 | 49 | **37** |  | 27.18 | 37.46 | 60.59 | **41.74** |  | 3.43 | 3.93 | 5.74 | **4.37** |
| L16 | 29 | 37 | 47 | **38** |  | 14.29 | 37.95 | 45.84 | **32.69** |  | 2.90 | 3.34 | 5.38 | **3.87** |
| L17 | 30 | 37 | 46 | **38** |  | 29.01 | 37.26 | 51.65 | **39.31** |  | 3.60 | 4.63 | 5.26 | **4.50** |
| L18 | 27 | 37 | 46 | **37** |  | 18.36 | 18.41 | 44.26 | **27.01** |  | 3.38 | 4.50 | 4.74 | **4.21** |
| Mean | **29** | **38** | **49** | **39** |  | **22.89** | **34.46** | **52.78** | **36.71** |  | **3.36** | **4.27** | **5.96** | **4.53** |
| Minimum | **27** | **36** | **45** | **35** |  | **14.25** | **21.89** | **44.26** | **27.01** |  | **2.64** | **3.34** | **4.74** | **3.87** |
| Maximum | **32** | **44** | **56** | **44** |  | **33.71** | **44.46** | **61.64** | **45.56** |  | **4.01** | **5.61** | **8.11** | **5.91** |
| LSD p=0.05 water regime | **2.29** | | | |  | **2.63** | | | |  | **0.31** | | | |
| LSD p=0.05 genotype | **2.08** | | | |  | **3.66** | | | |  | **0.40** | | | |
| LSD p=0.05 water regime x genotype | **3.40** | | | | | **5.46** | | | | | **0.60** | | | |

Gen = Genotype, Fl = Days to 50% flowering, Ph = Plant height, Ll = Leaf length

Table 1b Means for leaf width, stem diameter, and chlorophyll content of 18 African Spider plant accessions across three water regimes over two seasons.

|  |  | **Lw** |  |  |  |  | **Sd** |  |  |  |  | **Spad** |  |  |
| --- | --- | --- | --- | --- | --- | --- | --- | --- | --- | --- | --- | --- | --- | --- |
| **Gen** | **SS** | **MS** | **WW** | **Mean** |  | **SS** | **MS** | **WW** | **Mean** |  | **SS** | **MS** | **WW** | **Mean** |
| L1 | 4.56 | 5.06 | 6.73 | **5.45** |  | 3.08 | 3.78 | 6.36 | **4.41** |  | 30.23 | 33.10 | 41.34 | **34.89** |
| L2 | 3.56 | 5.45 | 6.80 | **5.27** |  | 2.68 | 4.54 | 7.01 | **4.74** |  | 28.46 | 34.70 | 40.40 | **34.52** |
| L3 | 4.56 | 7.16 | 10.20 | **7.31** |  | 4.05 | 6.24 | 9.00 | **6.43** |  | 30.65 | 34.76 | 41.98 | **35.80** |
| L4 | 4.00 | 4.78 | 8.64 | **5.81** |  | 3.44 | 4.35 | 7.20 | **5.00** |  | 31.55 | 33.71 | 40.88 | **35.38** |
| L5 | 4.18 | 5.11 | 8.98 | **6.09** |  | 3.54 | 4.99 | 7.85 | **5.46** |  | 28.53 | 34.49 | 41.89 | **34.97** |
| L6 | 4.08 | 5.14 | 6.95 | **5.39** |  | 3.96 | 4.04 | 6.75 | **4.92** |  | 28.93 | 34.84 | 40.09 | **34.62** |
| L7 | 3.98 | 5.61 | 7.11 | **5.57** |  | 3.83 | 4.26 | 6.60 | **4.90** |  | 29.38 | 36.50 | 39.05 | **34.98** |
| L8 | 4.20 | 6.10 | 8.93 | **6.41** |  | 3.76 | 5.14 | 7.50 | **5.47** |  | 29.71 | 35.13 | 42.45 | **35.76** |
| L9 | 4.75 | 5.18 | 8.30 | **6.08** |  | 2.79 | 4.33 | 6.66 | **4.59** |  | 30.86 | 33.50 | 40.60 | **34.99** |
| L10 | 4.53 | 5.86 | 6.80 | **5.73** |  | 2.84 | 4.15 | 5.95 | **4.31** |  | 29.94 | 33.79 | 37.78 | **33.84** |
| L11 | 3.36 | 3.96 | 8.21 | **5.18** |  | 3.99 | 3.80 | 7.25 | **5.01** |  | 32.14 | 34.73 | 39.86 | **35.58** |
| L12 | 3.31 | 4.78 | 7.11 | **5.07** |  | 2.84 | 3.94 | 5.60 | **4.13** |  | 27.78 | 34.05 | 40.03 | **33.95** |
| L13 | 4.16 | 5.89 | 7.14 | **5.73** |  | 3.00 | 4.34 | 7.14 | **4.83** |  | 30.48 | 34.83 | 38.50 | **34.60** |
| L14 | 5.15 | 5.84 | 7.71 | **6.23** |  | 3.15 | 4.43 | 6.78 | **4.79** |  | 32.51 | 36.88 | 43.33 | **37.57** |
| L15 | 4.76 | 5.26 | 7.46 | **5.83** |  | 2.73 | 4.40 | 6.71 | **4.61** |  | 31.49 | 35.46 | 40.21 | **35.72** |
| L16 | 3.70 | 4.88 | 6.81 | **5.13** |  | 2.24 | 4.05 | 5.35 | **3.88** |  | 31.14 | 32.59 | 39.44 | **34.39** |
| L17 | 3.90 | 5.88 | 7.16 | **5.65** |  | 3.21 | 5.08 | 6.91 | **5.07** |  | 28.91 | 34.38 | 42.73 | **35.34** |
| L18 | 4.05 | 5.38 | 6.39 | **5.27** |  | 2.55 | 3.13 | 4.86 | **3.51** |  | 29.94 | 36.08 | 38.26 | **34.76** |
| Mean | **4.16** | **5.41** | **7.64** | **5.73** |  | **3.20** | **4.39** | **6.75** | **4.78** |  | **30.15** | **34.64** | **40.49** | **35.09** |
| Minimum | 3.31 | 3.96 | 6.39 | 5.07 |  | 2.24 | 3.13 | 4.86 | 3.51 |  | 27.78 | 32.59 | 37.78 | 33.84 |
| Maximum | 5.15 | 7.16 | 10.20 | 7.31 |  | 4.05 | 6.24 | 9.00 | 6.43 |  | 32.51 | 36.88 | 43.33 | 37.57 |
| LSD p=0.05 water regime | **0.45** | | | |  | **0.36** | | | | | **1.10** | | | |
| LSD p=0.05 genotype | **0.51** | | | | | **0.49** | | | | | **1.55** | | | |
| LSD p=0.05 water regime x genotype | **0.78** | | | |  | **0.71** | | | |  | **2.30** | | | |

Gen = Genotype, Lw = Leaf width, Sd = Stem diameter, Spad = Chlorophyll content

Table 1 c Means for relative water content, net photosynthesis rate, and stomatal conductance of 18 African Spider plant accessions across three water regimes over two seasons.

|  |  | **Rwc** |  |  |  |  | **Photo** |  |  |  |  | **Cond** |  |  |
| --- | --- | --- | --- | --- | --- | --- | --- | --- | --- | --- | --- | --- | --- | --- |
| **Gen** | **SS** | **MS** | **WW** | **Mean** |  | **SS** | **MS** | **WW** | **Mean** |  | **SS** | **MS** | **WW** | **Mean** |
| L1 | 33.46 | 53.34 | 73.03 | **53.28** |  | 47.50 | 60.18 | 71.76 | **59.81** |  | 0.1008 | 0.1265 | 0.1796 | **0.1356** |
| L2 | 36.95 | 42.31 | 66.98 | **48.75** |  | 42.48 | 58.94 | 70.89 | **57.44** |  | 0.1345 | 0.0942 | 0.2148 | **0.1478** |
| L3 | 48.83 | 62.93 | 88.00 | **66.59** |  | 52.57 | 64.64 | 89.08 | **68.76** |  | 0.1703 | 0.1187 | 0.2931 | **0.1940** |
| L4 | 35.22 | 47.85 | 73.18 | **52.08** |  | 44.98 | 61.86 | 78.91 | **61.92** |  | 0.1182 | 0.1334 | 0.2547 | **0.1688** |
| L5 | 49.16 | 61.70 | 87.43 | **66.10** |  | 56.74 | 65.15 | 84.97 | **68.95** |  | 0.1212 | 0.1629 | 0.2847 | **0.1896** |
| L6 | 43.14 | 63.42 | 77.00 | **61.19** |  | 44.35 | 54.82 | 82.74 | **60.64** |  | 0.1252 | 0.1724 | 0.2787 | **0.1921** |
| L7 | 40.04 | 45.80 | 56.75 | **47.53** |  | 41.86 | 59.85 | 74.83 | **58.85** |  | 0.1111 | 0.1727 | 0.2507 | **0.1782** |
| L8 | 36.42 | 48.05 | 73.95 | **52.81** |  | 46.41 | 64.13 | 73.80 | **61.45** |  | 0.1117 | 0.1434 | 0.2184 | **0.1578** |
| L9 | 42.33 | 52.80 | 75.60 | **56.91** |  | 42.60 | 57.80 | 73.00 | **57.80** |  | 0.1594 | 0.1268 | 0.2495 | **0.1786** |
| L10 | 37.43 | 51.29 | 70.58 | **53.10** |  | 41.11 | 61.07 | 69.93 | **57.37** |  | 0.1180 | 0.1544 | 0.1625 | **0.1450** |
| L11 | 38.43 | 53.27 | 77.62 | **56.44** |  | 45.27 | 61.81 | 74.06 | **60.38** |  | 0.1143 | 0.1784 | 0.2451 | **0.1793** |
| L12 | 31.84 | 52.21 | 77.06 | **53.70** |  | 41.76 | 59.58 | 72.97 | **58.10** |  | 0.1031 | 0.1679 | 0.2274 | **0.1661** |
| L13 | 34.45 | 44.31 | 71.71 | **50.16** |  | 41.11 | 56.94 | 73.41 | **57.15** |  | 0.1078 | 0.1838 | 0.2422 | **0.1779** |
| L14 | 41.32 | 53.10 | 81.96 | **58.79** |  | 46.11 | 59.37 | 77.15 | **60.88** |  | 0.1157 | 0.1885 | 0.2908 | **0.1983** |
| L15 | 36.68 | 54.11 | 72.79 | **54.53** |  | 43.80 | 56.54 | 74.85 | **58.40** |  | 0.1131 | 0.1058 | 0.2058 | **0.1416** |
| L16 | 41.50 | 57.45 | 72.27 | **57.07** |  | 45.95 | 60.95 | 69.74 | **58.88** |  | 0.1184 | 0.2050 | 0.1504 | **0.1579** |
| L17 | 34.70 | 57.03 | 76.97 | **56.23** |  | 41.23 | 58.45 | 74.00 | **57.89** |  | 0.1387 | 0.1794 | 0.2120 | **0.1767** |
| L18 | 43.78 | 55.53 | 65.91 | **55.07** |  | 37.77 | 54.92 | 71.72 | **54.80** |  | 0.1017 | 0.0866 | 0.1971 | **0.1285** |
| Mean | **39.20** | **53.14** | **74.38** | **55.57** |  | **44.64** | **59.83** | **75.43** | **59.97** |  | **0.1213** | **0.1500** | **0.2310** | **0.1674** |
| Minimum | 31.84 | 42.31 | 56.75 | 48.75 |  | 37.77 | 54.92 | 69.74 | 54.80 |  | 0.1008 | 0.0866 | 0.1504 | 0.1285 |
| Maximum | 49.16 | 63.42 | 88.00 | 66.59 |  | 56.74 | 65.15 | 89.08 | 68.95 |  | 0.1703 | 0.2050 | 0.2931 | 0.1983 |
| LSD p= 0.05 water regime | **0** | | | |  | **1.39** | | | | | **0.02** | | | |
| LSD p= 0.05 genotype | **0** | | | | | **2.17** | | | | | **0** | | | |
| LSD p= 0.05 water regime x genotype | **0** | | | | | **3.16** | | | | | **0.01** | | | |

Gen = Genotype, Rwc = Relative water content, Photo= Net photosynthesis rate, Cond = Stomatal conductance

Table 1d Means for transpiration rate, number of leaves, and leaf yield of 18 African Spider plant accessions across three water regimes over two seasons.

|  |  | **Trans** |  |  |  |  | **Nl** |  |  |  |  | **Ly** |  |  |
| --- | --- | --- | --- | --- | --- | --- | --- | --- | --- | --- | --- | --- | --- | --- |
| **Gen** | **SS** | **MS** | **WW** | **Mean** |  | **SS** | **MS** | **WW** | **Mean** |  | **SS** | **MS** | **WW** | **Mean** |
| L1 | 0.0093 | 0.0138 | 0.0184 | **0.0138** |  | 32 | 46 | 79 | **52** |  | 24.89 | 31.34 | 64.19 | **40.14** |
| L2 | 0.0092 | 0.0115 | 0.0162 | **0.0123** |  | 11 | 33 | 53 | **32** |  | 3.43 | 11.98 | 37.60 | **17.67** |
| L3 | 0.0094 | 0.0131 | 0.0211 | **0.0145** |  | 44 | 56 | 126 | **75** |  | 26.99 | 46.49 | 131.19 | **68.22** |
| L4 | 0.0110 | 0.0126 | 0.0178 | **0.0138** |  | 16 | 39 | 76 | **44** |  | 10.97 | 19.04 | 101.67 | **43.89** |
| L5 | 0.0114 | 0.0134 | 0.0206 | **0.0151** |  | 37 | 49 | 131 | **72** |  | 19.06 | 59.27 | 118.78 | **65.70** |
| L6 | 0.0091 | 0.0122 | 0.0187 | **0.0133** |  | 20 | 44 | 125 | **63** |  | 5.81 | 14.15 | 112.42 | **44.13** |
| L7 | 0.0087 | 0.0135 | 0.0197 | **0.0140** |  | 22 | 51 | 112 | **62** |  | 3.20 | 27.80 | 97.50 | **42.83** |
| L8 | 0.0085 | 0.0141 | 0.0175 | **0.0134** |  | 25 | 55 | 94 | **58** |  | 11.45 | 39.31 | 72.26 | **41.01** |
| L9 | 0.0116 | 0.0121 | 0.0172 | **0.0136** |  | 14 | 33 | 62 | **36** |  | 8.80 | 18.33 | 40.00 | **22.38** |
| L10 | 0.0091 | 0.0140 | 0.0170 | **0.0134** |  | 17 | 44 | 53 | **38** |  | 4.05 | 17.95 | 26.57 | **16.19** |
| L11 | 0.0092 | 0.0111 | 0.0174 | **0.0126** |  | 18 | 36 | 58 | **37** |  | 5.67 | 34.87 | 71.69 | **37.41** |
| L12 | 0.0088 | 0.0107 | 0.0163 | **0.0119** |  | 13 | 26 | 52 | **30** |  | 3.74 | 6.96 | 35.66 | **15.45** |
| L13 | 0.0083 | 0.0131 | 0.0173 | **0.0129** |  | 16 | 31 | 53 | **33** |  | 4.98 | 12.21 | 45.17 | **20.79** |
| L14 | 0.0097 | 0.0134 | 0.0200 | **0.0144** |  | 24 | 76 | 113 | **71** |  | 9.26 | 31.25 | 72.49 | **37.67** |
| L15 | 0.0082 | 0.0099 | 0.0146 | **0.0109** |  | 15 | 53 | 79 | **49** |  | 5.33 | 19.19 | 59.71 | **28.08** |
| L16 | 0.0099 | 0.0129 | 0.0171 | **0.0133** |  | 26 | 38 | 65 | **43** |  | 15.07 | 20.10 | 49.21 | **28.13** |
| L17 | 0.0095 | 0.0112 | 0.0174 | **0.0127** |  | 25 | 44 | 80 | **50** |  | 12.02 | 24.43 | 58.18 | **31.54** |
| L18 | 0.0082 | 0.0110 | 0.0158 | **0.0117** |  | 14 | 26 | 54 | **31** |  | 3.33 | 12.68 | 32.54 | **16.18** |
| Mean | **0.0094** | **0.0124** | **0.0178** | **0.0132** |  | **22** | **43** | **81** | **49** |  | **9.89** | **24.85** | **68.16** | **34.30** |
| Minimum | 0.0082 | 0.0099 | 0.0146 | 0.0109 |  | 11 | 26 | 52 | 30 |  | 3.20 | 6.96 | 26.57 | 15.45 |
| Maximum | 0.0116 | 0.0141 | 0.0211 | 0.0151 |  | 44 | 76 | 131 | 75 |  | 26.99 | 59.27 | 131.19 | 68.22 |
| LSD p = 0.05water regime | **0** | | | |  | **1.93** | | | | | **2.16** | | | |
| LSD p = 0.05 genotype | **0** | | | |  | **3.30** | | | | | **2.04** | | | |
| LSD p = 0.05 water regime x genotype | **0** | | | |  | **4.77** | | | | | **3.27** | | | |

Gen = Genotype, Trans = Transpiration rate, Nl = Number of leaves per plant, Ly = Leaf yield

Table 1e Means for proline content of 18 African Spider plant accessions across three water regimes over two seasons.

|  |  | **PRO** |  |  |
| --- | --- | --- | --- | --- |
| **Gen** | **SS** | **MS** | **WW** | **Mean** |
| L1 | 355.34 | 176.44 | 46.25 | 192.68 |
| L2 | 323.38 | 185.28 | 42.88 | 183.85 |
| L3 | 356.10 | 182.21 | 50.65 | 196.32 |
| L4 | 349.11 | 202.31 | 41.27 | 197.56 |
| L5 | 362.28 | 210.48 | 51.45 | 208.07 |
| L6 | 345.63 | 169.39 | 44.51 | 186.51 |
| L7 | 367.03 | 175.29 | 75.85 | 206.06 |
| L8 | 325.80 | 182.33 | 49.65 | 185.93 |
| L9 | 362.23 | 196.43 | 48.25 | 202.30 |
| L10 | 345.72 | 189.73 | 47.03 | 194.16 |
| L11 | 338.60 | 191.12 | 40.06 | 189.93 |
| L12 | 345.27 | 184.25 | 44.55 | 191.36 |
| L13 | 356.44 | 189.18 | 45.28 | 196.97 |
| L14 | 369.44 | 184.54 | 46.01 | 200.00 |
| L15 | 354.20 | 183.83 | 49.14 | 195.72 |
| L16 | 312.53 | 200.02 | 44.57 | 185.71 |
| L17 | 369.52 | 180.58 | 53.60 | 201.23 |
| L18 | 373.38 | 203.37 | 48.70 | 208.48 |
| Mean | **350.67** | **188.15** | **48.32** | **195.71** |
| Minimum | 312.53 | 169.39 | 40.06 | 183.85 |
| Maximum | 373.38 | 210.48 | 75.85 | 208.48 |
| LSD p= 0.05 water regime | **18.86** | | | |
| LSD p= 0.05 genotype | **19.31** | | | |
| LSD p= 0.05 water regime x genotype | **30.59** | | | |

Gen = Genotype, Pro = Proline content
